# Automatic Extraction of Nuclei Centroids of Mouse Embryonic Cells from Fluorescence Microscopy Images

**DOI:** 10.1371/journal.pone.0035550

**Published:** 2012-05-08

**Authors:** Md. Khayrul Bashar, Koji Komatsu, Toshihiko Fujimori, Tetsuya J. Kobayashi

**Affiliations:** 1 Institute of Industrial Science, The University of Tokyo, Tokyo, Japan; 2 Division of Embryology, National Institute for Basic Biology, Okazaki, Aichi, Japan; 3 Division of Embryology, National Institute for Basic Biology, Okazaki, Aichi, Japan; 4 Institute of Industrial Science, The University of Tokyo, Tokyo, Japan; Università degli Studi di Milano, Italy

## Abstract

Accurate identification of cell nuclei and their tracking using three dimensional (3D) microscopic images is a demanding task in many biological studies. Manual identification of nuclei centroids from images is an error-prone task, sometimes impossible to accomplish due to low contrast and the presence of noise. Nonetheless, only a few methods are available for 3D bioimaging applications, which sharply contrast with 2D analysis, where many methods already exist. In addition, most methods essentially adopt segmentation for which a reliable solution is still unknown, especially for 3D bio-images having juxtaposed cells. In this work, we propose a new method that can directly extract nuclei centroids from fluorescence microscopy images. This method involves three steps: (i) Pre-processing, (ii) Local enhancement, and (iii) Centroid extraction. The first step includes two variations: first variation (Variant-1) uses the whole 3D pre-processed image, whereas the second one (Variant-2) modifies the preprocessed image to the candidate regions or the candidate hybrid image for further processing. At the second step, a multiscale cube filtering is employed in order to locally enhance the pre-processed image. Centroid extraction in the third step consists of three stages. In Stage-1, we compute a local characteristic ratio at every voxel and extract local maxima regions as candidate centroids using a ratio threshold. Stage-2 processing removes spurious centroids from Stage-1 results by analyzing shapes of intensity profiles from the enhanced image. An iterative procedure based on the nearest neighborhood principle is then proposed to combine if there are fragmented nuclei. Both qualitative and quantitative analyses on a set of 100 images of 3D mouse embryo are performed. Investigations reveal a promising achievement of the technique presented in terms of average sensitivity and precision (i.e., 88.04% and 91.30% for Variant-1; 86.19% and 95.00% for Variant-2), when compared with an existing method (86.06% and 90.11%), originally developed for analyzing *C. elegans* images.

## Introduction

The reliable extraction of nuclei centroids from cells using three-dimensional (3D) digital images is an important task in various biological studies. For example, understanding embryogenesis requires the tracking of cell nuclei that actively divide and move in the embryo [1]. Accurate cancer diagnosis or the understanding of the healing process in the damaged tissue also requires analysis of velocities and accelerations of cell nuclei of migrating cells [2]. Recent advances in time-lapse fluorescence microscopy imaging have provided an important tool for studying the dynamics of cell-nuclei under different experimental conditions.

Most methods in the 3D analysis of cells from fluorescence images are manual and/or interactive. Schnabel *et al.* proposed a software (SIMI Biocell software) for lineage analysis, which implements a 3D interactive method for manually identifying cell-nuclei of *C. elegans* [3]. Parfitt *et al.* used the same technique to regulate lineage allocation in the early mouse embryo [4]. Although these methods improve cell analysis, manual cell marking by clicking computer-mouse is time-consuming and error-prone. In recent years, increasing efforts in developing automated methods for the extraction of cell nuclei from 3D/4D images have been made [2], [5]. However, most of these methods perform segmentation followed by centroid extraction [1], [6], [7], [8]. The final outcome is therefore strongly dependent on accuracy of the segmentation procedures. However, typical segmentation methods [9] do not work well with low contrast fluorescence images. Although a few advanced methods [5] have been attempted, the accurate segmentation of cell nuclei is still an issue to be resolved, especially in the case of touching cells that are frequently observed during mouse embryogenesis. Hamahashi *et al.* used local entropy to characterize smooth textural properties of cell nuclei of *C. elegans* in differential interference contrast (DIC) images and claim to have achieved successful detection up to the 24-cell stage [1]. However, their method seems inapplicable directly to fluorescence images because of lower texture contrast in fluorescence images. Keller *et al.* analyzed the embryogenesis of zebra fish by using specially designed digital scanned laser light sheet fluorescence microscopy (DSLM) [8]. Their method applies recursive segmentation based on shapes and internal structures of cells. A good outcome from segmentation can be obtained because of high signal to noise ratio (SNR) of the DSIM images. However, the mouse embryos are quite different from those of the zebra fish. In zebra fish, cells grow in a thin peripheral layer that covers transparent internal materials. Therefore, the developed method, which is specific to certain embryo characteristics and special imaging technique, may not be applicable to usual fluorescence images for mouse embryos. Recently, Oleh *et al.* proposed a level–set–based technique for the segmentation and tracking cell nuclei of 2D human HeLa cells from fluorescence microscopy images [6]. Although this method claimed to have an improved tracking performance, it was not tested with mouse embryo images.

An alternative way is therefore the direct extraction of nuclei-centroids. Bao *et al.* proposed one such method, which adopts a single–scale local filter and a fixed spatial distance for directly extracting nuclei centroids from the *C. elegans* embryo images [10]. However, compared to *C. elegans*, mouse embryonic cells have larger movements with variable nuclei sizes [11]. Therefore, the assumptions of using single scale and/or fixed spatial distance in Boa’s method seem insufficient for the analysis of mouse embryo images.

In this paper, we propose an efficient method to automate the detection of nuclei centroids in mouse embryos. Our method performs multiscale transformation and local maxima computation to detect nuclei centroids automatically in a set of 3D fluorescence microscope images. Profile shape analysis and iterative merging the nuclei fragments make the method suitable to extract nuclei centroids from juxtaposed or dividing cells irrespective of their sizes, shapes, or numbers. We applied the proposed method to mouse embryo images having 17 to 33 cells and found it effective in terms of nuclei detection.

## Materials and Methods

### Fluorescence Imaging Data

**Figure 1:**
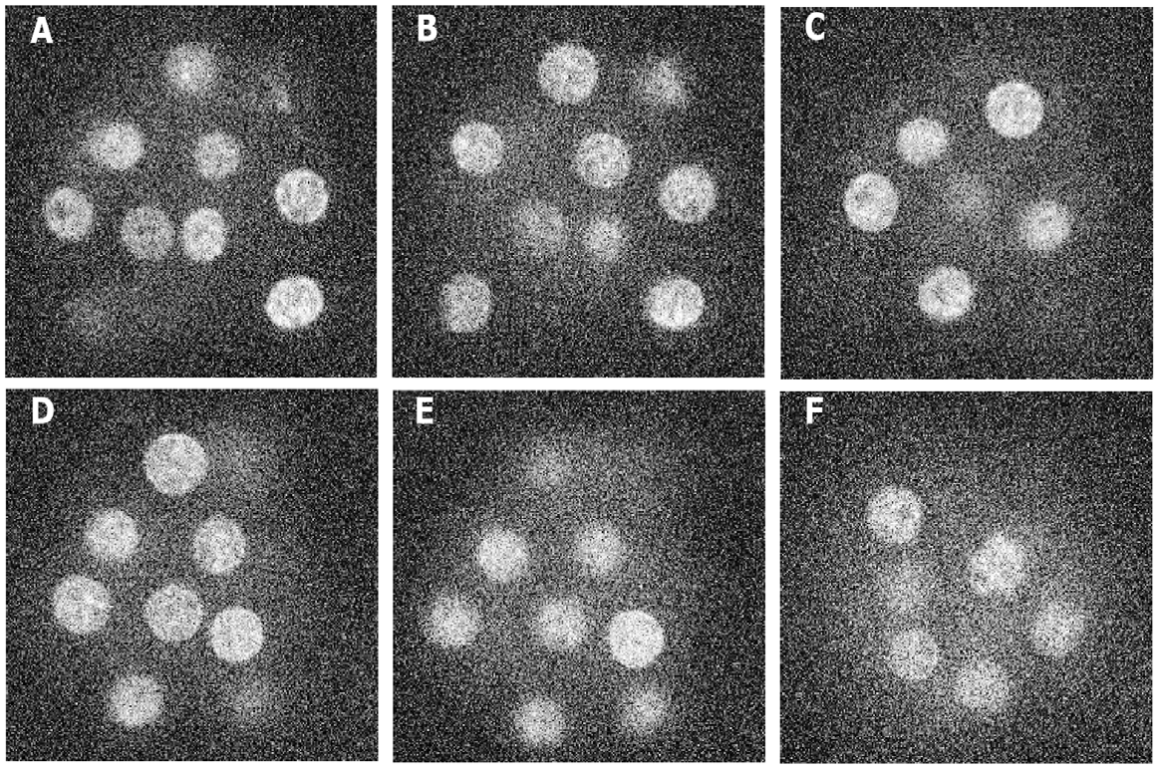
Histogram equalized 2D sample images from our image dataset. Sample images with (time point, z-slice) pairs at (A) (5,13), (B) (9,14), (c) (10,11), (D)(25,14), (E) (36,15), and (F) (83,18). Each image has dimension: 241×241 pixels and has voxel resolutions: 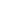
x = 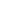
y = 0.385 and 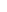
z = 3 microns.

Mouse embryo images are captured by fluorescence microscopy equipped with confocal system. The nuclei are labeled with histone H2EGFP [12]. The microscope used for the mouse embryo image-set is a Leica DMIRBE and a spinning confocal system (CSU-10, Yokogawa, Tokyo) using 488 nm laser. Each of the original voxels has a resolution of 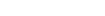 and 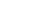 in the x-, y-, and z-directions. At a time instant, 28 cross-sectional images span over a whole embryo in the z-direction. These images construct a 3D volume image by stacking sequentially. We perform cubic interpolation that generates 224 slices, which is eight times more than that of the original slices. This results in approximately isotropic voxels of resolution 0.385×0.385×0.375^3^. The details of the experimental and imaging settings can be obtained from [12]. In our experiment, we chose a dataset of 100 3D stack images, which were indexed from t1 to t100. Each image has 261×261×224 pixels. Image set has the temporal resolution of 10 minutes and contains 17 to 33 mouse embryo cells. The whole image set can be divided into two temporal slots based on the number of cells. In the first slot (t1 to t66), cells remain constant in number, while in the second slot (t67 to t100), their number increases due to cell division. For the convenience in representation, we consider these slots as the ‘silent’ and ‘active’ states, respectively. Figure 1 shows a set of contrast-enhanced sample 2D images, while File S2 shows an enhanced video clip that corresponds to the time point t10 of our dataset. Windows media player can be used to visualize this clip. Please refer to File S1 for detail procedure.

In our work, experiments on mice were performed in order to extract embryos for fluorescence imaging. All animal care procedures were carried out pursuant to the Guidelines for Animal Experimentation of the National Institute for Basic Biology and National Institute for Physiological Sciences, Okazaki, Japan.The animal experiments were approved by "the Institutional Animal Care and Use Committee of National Institutes of Natural Sciences". In this approval, Koji Komatsu and Toshihiko Fujimori are included.

### Creation of Ground Truth Data

Ground–truth (GT) data for the nuclei centroids are created by manually marking the approximate centroids of mouse embryonic cells in 3D fluorescence images. First of all, we generate preprocessed images by Gaussian smoothing and median filtering with appropriate interpolation. Preprocessed images are displayed using a visualization software PLUTO [13]. With the help of threshold setting in the visualizing software, we can generate approximate segmentation of cell-nuclei from each 3D image. We can also track nuclei sizes and shapes by examining 2D slices in the z-direction. The centroids of all available nuclei in an image were marked by mouse clicking on appropriate slices after manual justification. Two observers marked centroid coordinates of nuclei from a total of 100 3D images. We thus obtain ground truth (GT) centroids for the given dataset.

### Proposed Method

#### Overview

**Figure 2:**
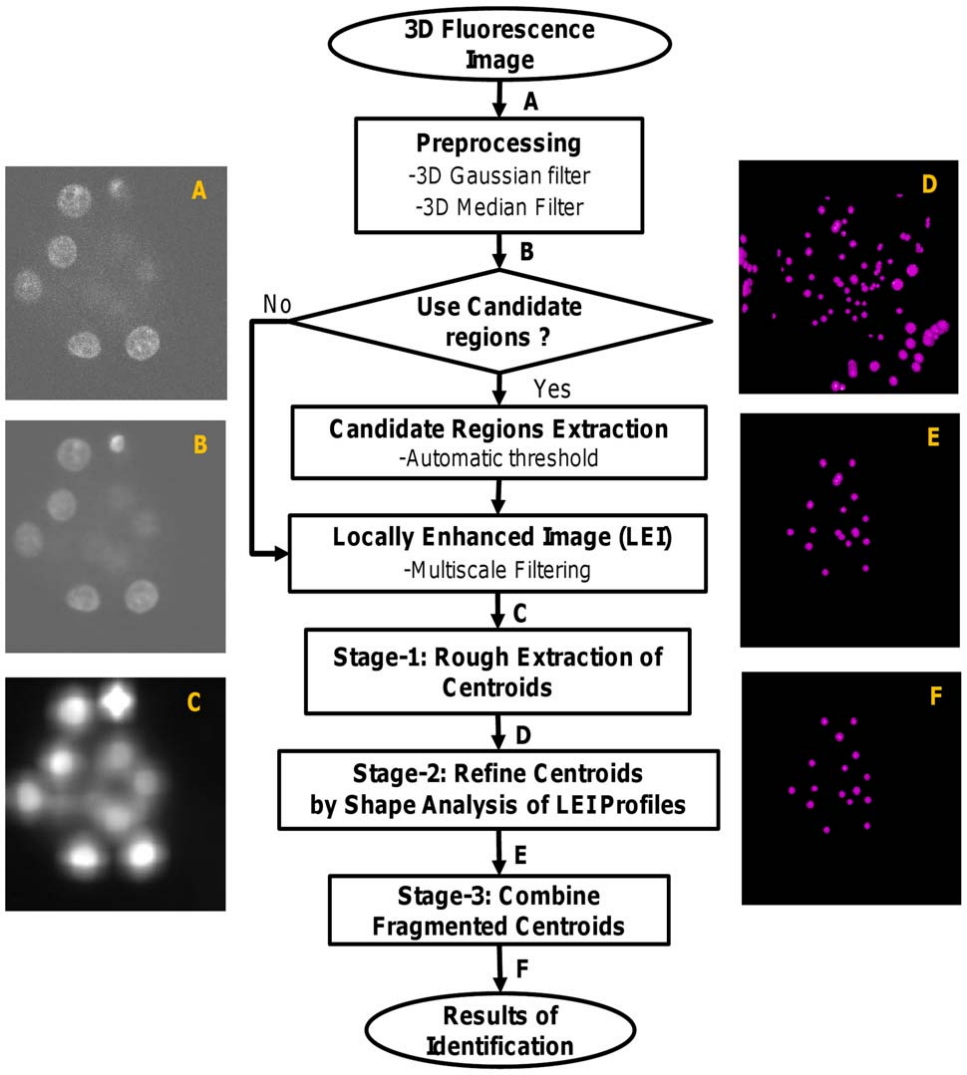
Flow diagram of proposed method. Detailed block diagrams of our proposed methods. Two dimensional (2D) version of the original 3D (A) Input image, (B) Pre-processed image, (C) Locally enhanced image (LEI) image; 2D version of the volume rendered images as (D) result of rough centroid extraction, (E) refined result after local shape analysis of LEI profiles, and (F) final result of centroid extraction after combining fragmented nuclei.

We propose a novel method for the automated extraction of nuclei centroids from fluorescence microscopy images. Two variations of the proposed method were achieved by modifying the output of the preprocessing step (to be explained below) keeping the other steps intact. Variant-1 uses the whole preprocessed image without any modification, while Variant-2 modifies the preprocessed image to obtain candidate regions or the candidate hybrid image for further processing. An overview of our method is shown in Fig. 2. An input image (Fig. 2 A) is first preprocessed with a 3D Gaussian filter followed by a 3D median filter to minimize the effects of high–frequency and impulsive noises. This image (Fig. 2 B) as a whole or its approximate object regions can be used for further processing. Candidate object regions can be obtained by an automatic threshold technique [14]. A multiscale filtering (MSF) [15] using a 3D cubic filter is then performed on all voxels of the preprocessed image (Variant-1) or its candidate regions (Variant-2). This procedure enhances image objects (i.e., nuclei) by locally maximizing filtering responses. A three stage procedure then follows. Stage-1 computes candidate local maxima as a set of binary clusters or regions from enhanced images (Fig. 2 C). These regions roughly indicate nuclei centroids in cells (Fig. 2 D). However, some spurious regions due to unavoidable noise components may be included in these regions. Therefore, a second stage (Stage-2) follows that exploits shapes of intensity profiles in the enhanced image and removes some unexpected regions from Stage-1 results. However, Stage-2 outcome (Fig. 2 E) may contain fragmented nuclei that may be due to intra-nuclear inhomogeneity. A third stage (Stage-3) is applied to combine fragmented nuclei (Fig. 2 F).

#### Pre-processing

Fluorescence images captured by microscopy have noises and other artifacts. We, therefore, apply a two-step procedure for noise reduction: lowpass filtering followed by median filtering. A 3D Gaussian filter of size (5×5×3) pixels with 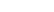 is used to reduce high–frequency noise. The half-length of the filter in the r-*th* direction is computed using 
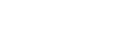
 where 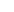 is the voxel resolution. With the given resolution of image voxels i.e., 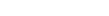 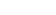 these becomes 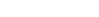 and 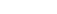 voxels, which ultimately give the filter size of 5×5×3. The background image is not uniform because of fluorescence effects. Sometimes, incorrect parameter settings may introduce partial occlusion of image objects, including unexpected discrete noises. A 3D median filter of size (3×3×3) pixels is also used to remove impulsive noise. A cubic interpolation is then performed to obtain approximately isotropic voxels for further processing.

**Figure 3:**
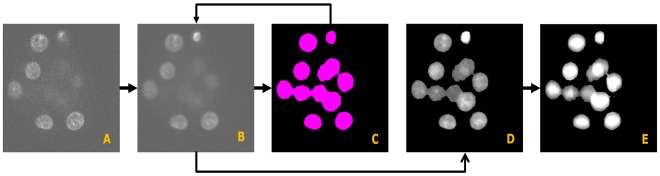
An Example of processing results for candidate regions and enhanced image. Two dimensional (2D) version of the original 3D (A) Input image, (B) Preprocessed image, (C) Candidate masks, (D) Candidate regions, and (E) Locally enhanced image (LEI).

*(a) Generation of Candidate Regions:* Although the processing of full 3D images is usual, the use of candidate regions may bring benefits in two ways. First, it saves processing time for large volume biological images. Secondly, it may improve the accuracy of local maxima computation; the accuracy usually falls for a noisy whole image that includes non-uniform backgrounds. However, the expected candidate regions should include all possible objects. We use Otsu’s global threshold method [14] to extract candidate regions from the preprocessed image. This method automatically creates binary masks in which nuclei regions are labeled ‘1’ and the rest are labeled ‘0’. The content of the preprocessed image corresponding to voxels having ‘1’ labels are retained to construct a hybrid image, which can be used for subsequent processing. Figure 3-(D) shows an example of the generated candidate regions. Experiment shows that despite having a degree of non-uniform illuminations in the imaging data, this method works well with the confocal fluorescence microscopy images.

For the sake of clarity, the remaining steps of our method and the relevant mathematical expressions will be described according to Variant-1, although a brief explanation regarding Variant-2 will be given wherever necessary.

#### Local enhancement by multiscale filtering

Since cell population in the embryo increases over time, the imaging technique sometimes fails to capture contrast between the object and background regions. Moreover, the power of emitted light to larger and smaller nuclei is not uniform even at a single time point. The local transformation of the pre-processed image is therefore an important step in our research. It brings benefits by smoothing object boundaries which facilitate computing stable local maxima that ultimately leads to the extraction of cell nuclei. The central region of a cell has higher luminance, which decreases gradually towards the cell/nuclei boundaries. To deal with these characteristics including the variable sizes of mouse embryonic cell nuclei, we propose a multiscale filtering that perform local optimization of multiple responses at every voxel. This involves the convolution of 3D images with 3D cube filters. For ease of computation, we consider separable one–dimensional filters in three orthogonal directions [16]. We therefore scan an image stack (volume) three times, one dimension at a time, with the result of the previous scan being used as the input for the next. If the filtering responses are denoted by 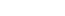 we can obtain locally enhanced image (LEI), 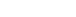 as an optimal response image by
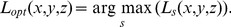
(1)


If 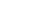 is a 3D preprocessed image and 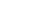 is a cubic filter, then the multiscale local signal can be computed from
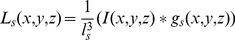
(2)

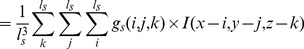
(3)

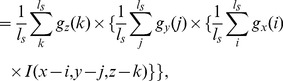
(4)where the filter length in our multiscale framework is given by 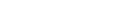 for 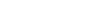 and 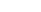 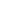 can be computed by 
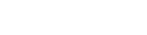
 where 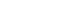
%×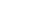 and 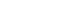
%×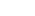 are percentages of the diameter (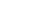) of the largest nucleus, empirically fixed by observing the fluorescence image corresponding to the lowest time–point. We used the assumption of separability to obtain Eq. 4 from Eq. 3. The one–dimensional cubic filter *g*(*i*) is defined by



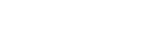



The result of the optimized filtering, 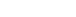 is used in the next step to compute the cell nuclei. Figure 3- (A–E) shows the sequential results of generating locally enhanced image by this method.

#### Centroid extraction

We propose three stages for the centroid extraction from 3D images. Stage-1 extracts candidate nuclei centroids; Stage-2 and Stage-3 refines the results of initial detection.

*Stage-1: Extraction of Candidate Nuclei Centroids:* Since central regions of nuclei have higher intensities that gradually fall towards nuclei boundaries, the local maxima of 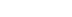 will correspond to the centroids of nuclei in fluorescence images. Computing local maxima for a 3D object ideally involves data investigation in many directions, which is time-consuming task. We propose below a simple method based on the local voxel-ratio measure, called the characteristic ratio (*R*). At every voxel, the ratio is computed by counting neighboring voxels having intensities smaller than or equal to the intensity of the central voxel in the cubic neighborhood *V*. A typical neighborhood is usually less than or equal to the smallest object in an image. To avoid spurious detection, its maximum size should be such that it can enclose only a single object (nucleus). We select (7×7×7) as a reasonable choice of a neighborhood. Finally, candidate local maxima are identified by threshold operation. If we assume a neighborhood of size 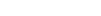 around a voxel 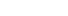 it can be defined as 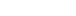  =  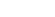 
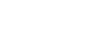
 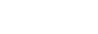 
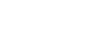
 Therefore, the characteristic ratio can be defined by
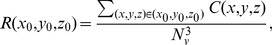
(5)where



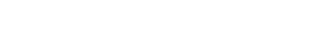



By using the discussed voxel-ratio above, we can extract centroid clusters image, 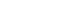 by
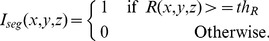
(6)


We perform connected component labeling on 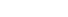 followed by the the averaging of the voxels coordinates of each component. This procedure generates candidate binary centroid image, 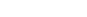 as Stage-1 output. Finally, we can also obtain Stage-1 label centroid image by labeling a spherical region around each estimated centroid. The systematic procedure regarding above detection is given below.

Algorithm for the extraction of candidate nuclei centroids.

- Input: Optimized local image, 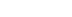.
- Output: Candidate centroid image, 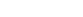.
- Initialize 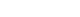 to zero and assume a ratio threshold, 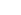

1. Select a small cube (*V*) (see definition above) of size (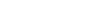) around each non-zero voxel of 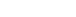

2. Let 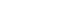 be the center of *V* and 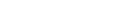.
3. Count voxels that satisfy the condition 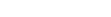 for all 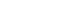

4. Compute the characteristic ratio, *R* using Eq. 5.
5. Assign 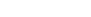 if 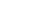 This is a binary image that contains nuclei central regions.
6. Continue above steps for all non-zero voxels in 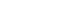

7. Perform connected component labeling of the binary centroid cluster image, 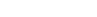

8. Compute centroids by averaging the coordinates of the voxels in each component. This will create a binary centroid image, 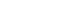

9. Create a label centroid image, 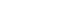 by labeling candidate centroids.

**Figure 4:**
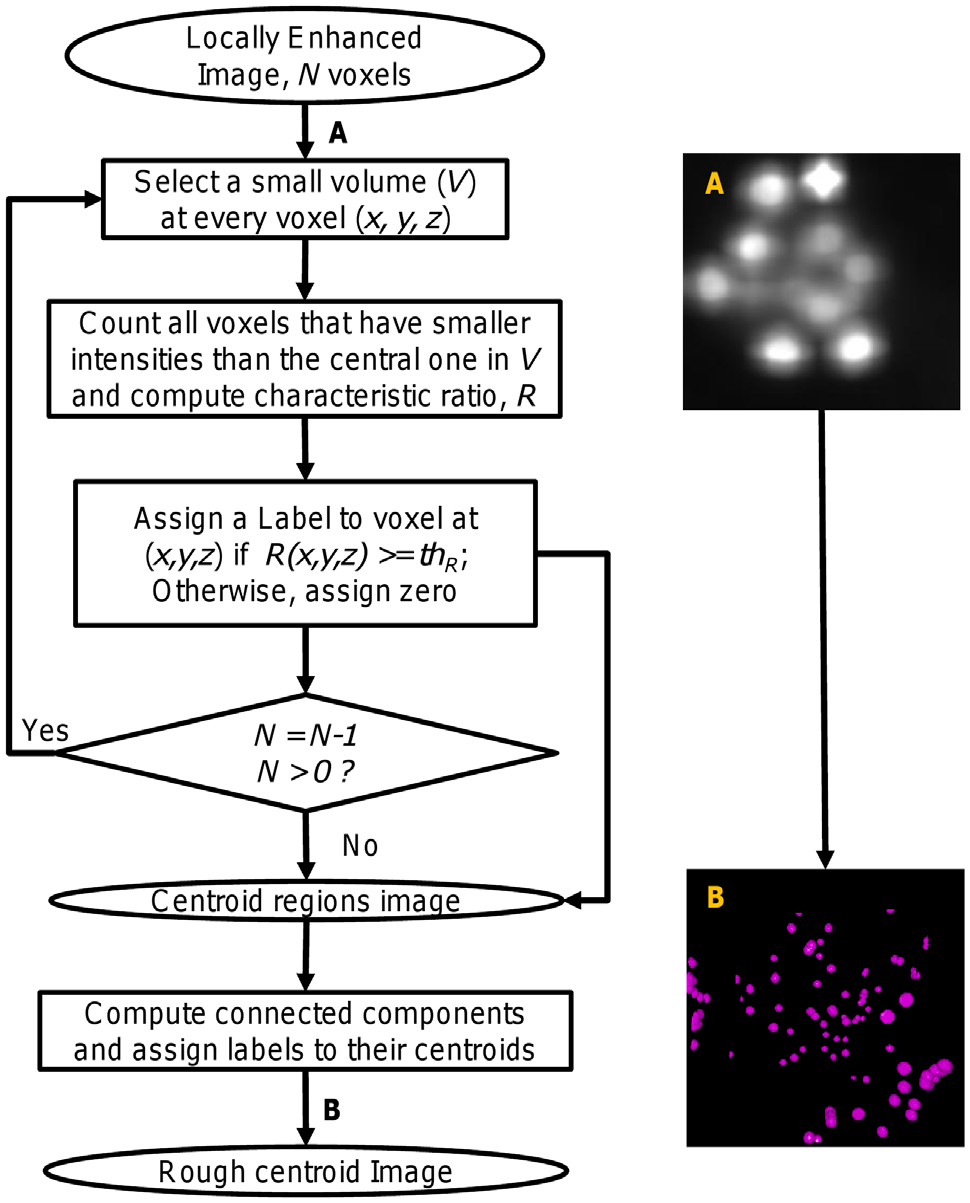
Procedure for extraction of candidate nuclei centroids (Stage-1). Block diagram shows how we obtain candidate centroids systematically from locally enhanced image. (A) Locally enhanced image (LEI), (B) Candidate centroids. Spherical color regions indicate centers of local maxima regions.

Figure 4 shows the block diagram of our proposed method for the rough extraction of nuclei centroids. This procedure roughly generates candidate centroids for a given 3D image. The ratio threshold above plays an important role in this stage. Usually, the smaller the threshold value the larger is the number of the estimated centroids and vice versa. For an ideal object, we expect a single maximum pixel, which will give a ratio threshold of 100%. However, practical situation is different due to the presence of noise and other artifacts. We empirically set a threshold value, 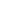 of 97% in the proposed method.

In the case of Variant-2, 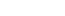 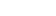 and 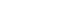 in Eqs. 1, 2, and 5 represent the hybrid images, which correspond to the preprocessed, multiscale filtered, and the optimal response images in Variant-1. Therefore, all processing related to above functions were performed only on the non-zero voxels of the relevent hybrid image in case of Variant-2 of the proposed method.

However, various micro-structures in the nuclei may produce several intra-nuclear maxima clusters including some spurious regions due to the noise or inhomogeneous distribution of intensities. This problem needs to be addressed before obtaining the final centroids.

*Stage-2: Refinement of Centroids by Profile Shape Analysis:* The over detection of the nuclei centroids in Stage-1 is mainly observed in the background- and boundary- regions. The processing in this stage identifies these undesired centroids and refines the detection results by analyzing shapes of the local profiles from LEI (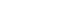) in three orthogonal directions. For a given profile *P*(*i*), we first define a label function *L*(*i*) that corresponds to the slopes at every discrete point of the profile.
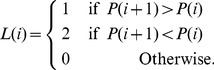
(7)


Based on the label function, we define a shape score, *S*, given by
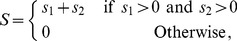
(8)where



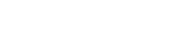


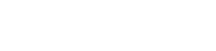
and 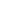 is the profile length, which is 70% of the largest nucleus diameter, selected empirically. Through above definition, we approximately measure the shape of nuclei without adopting any curve fitting technique. We also discard any partial nucleus or background regions by using above score. The above procedure is performed at each candidate centroids. If 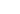 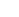 and 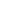 represent scores at a centroid 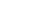 in three orthogonal directions, the final shape score (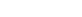) can be defined by



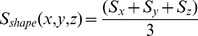
(9)Note that 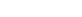 has a maximum value of 1.0, when all three orthogonal profiles have symmetric convex shapes with the consecutive positive and negative slopes. If symmetry and/or smoothness of the profile goes down, the score decreases gradually. For flat or linear profiles the score is always zero. We can thus obtain Stage-2 binary centroid image 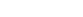 by removing false centroids from Stage-1 results by
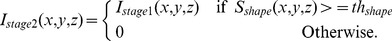
(10)


Therefore, Stage-2 results depend on the selection of the threshold value. A large value has to be chosen to remove false centroids that were detected at Stage-1. This stage performs a huge reduction of the false positives, especially for Variant-1 of our method. We empirically selected this threshold as 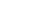 = 85%. The following is the systematic approach to perform refining work at this stage.

Algorithm for Stage-2 refining.

- Inputs: Optimized local signal, 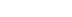 and Candidate centroid image, 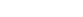.
- Output: Refined centroid image, 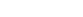.
- Assume a threshold value that represents the shape of the local intensity profile, 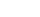.

1. Extract coordinates for all candidate centroid from 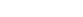

2. For each centroid, extract intensity profiles from 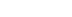 along *x*-, *y*-, and *z*-directions.
3. Compute profile shape score, 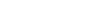 according to the criterion defined in Eq. 9
4. Remove the label for the centroid from 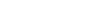 when 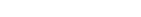.
5. Continue step- 2 to step- 4 for the remaining centroids.
6. Create Stage-2 binary centroid image 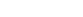 by assigning refined results from 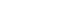.
7. Create Stage-2 label centroid image, 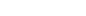 by labeling the centroids from 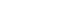.

**Figure 5:**
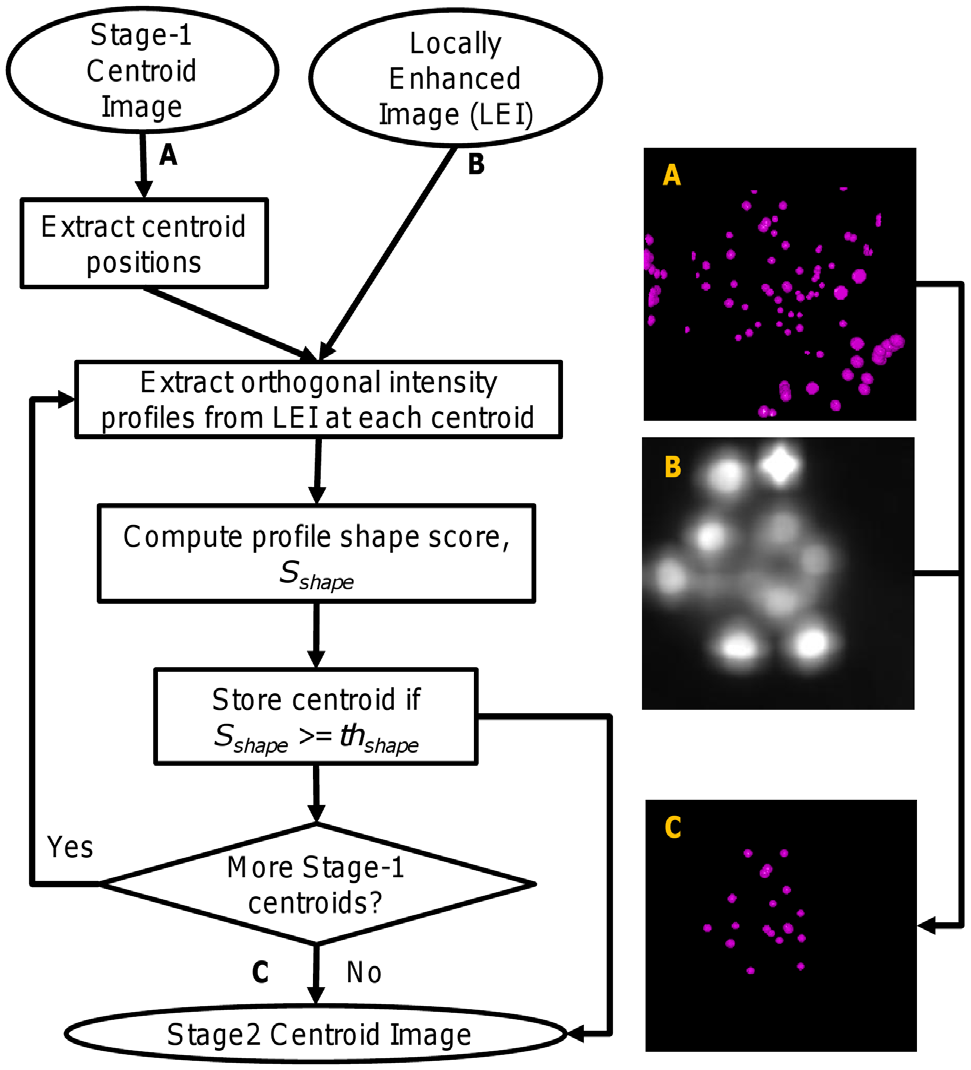
Procedure for refining the results of initial centroid detection (Stage-2). Block diagram shows the procedure for refining the results of Stage-1 detection. (A) Rough centroids, (B)Locally enhanced image (LEI), and (C) Stage-2 centroids after removing some false centroids in Stage-1.

Stage-2 refining removes most of the spurious centroids, which were detected in Stage-1. The processing steps described above are shown in Fig. 5.

**Figure 6:**
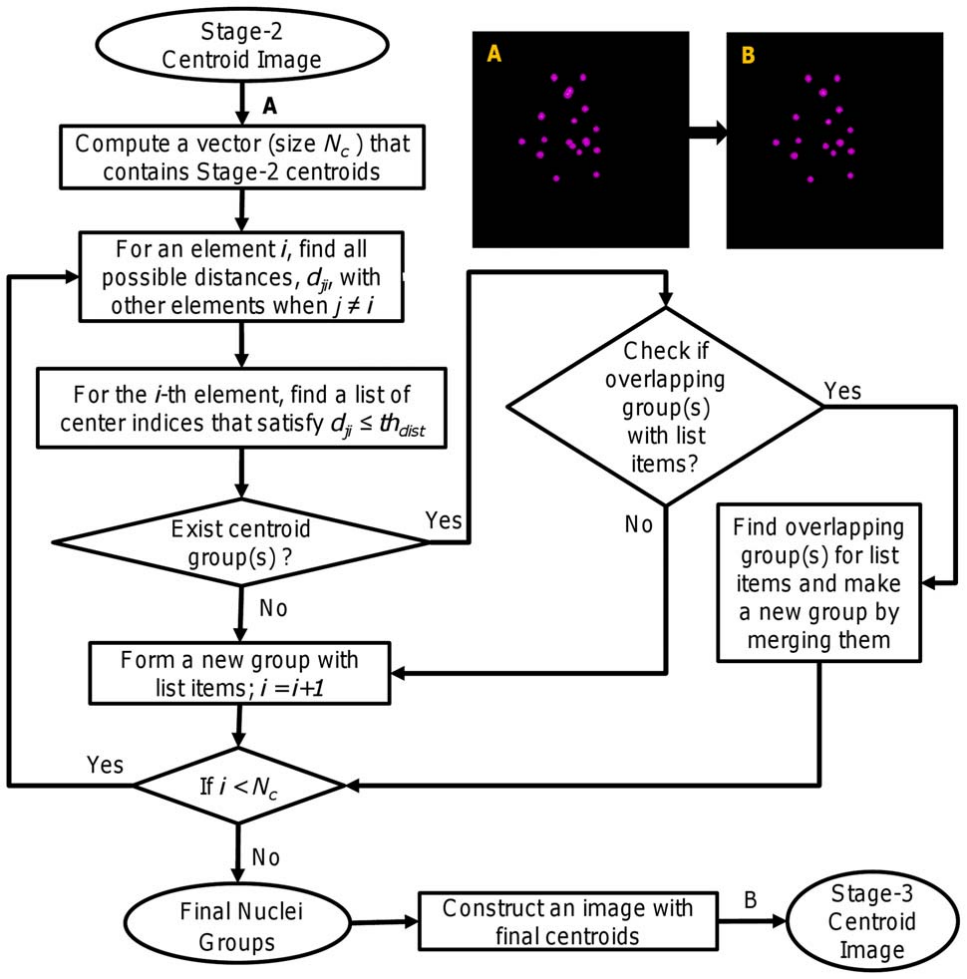
Procedure for combining fragmented nuclei (Stage-3). Schematic diagram shows the iterative grouping of fragmented nuclei if exist. (A) Stage-2 detection results, (B) Final nuclei centroids.

*Stage-3: Refinement of Stage-2 Results by Combining Fragmented Centroids:* Inhomogeneous intensity distribution due to intra-nuclear structures may produce multiple local maxima clusters during Stage-1 processing. Some of them may still remain even after Stage-2 processing. Stage-3 processing is necessary to combine fragmented nuclei (if any). An iterative procedure is proposed to combine fragmented nuclei. A threshold on the inter-region Euclidean distances is used in this procedure. If Stage-2 centroids are indexed by *i* and *j*, we may denote these distances by
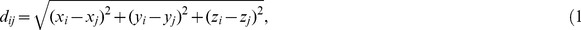
(11)where 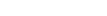 for 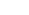 and 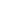 is the number of centroids after Stage-2 processing. Following is the systematic approach that is used in our research to combine fragmented nuclei. Figure 6 shows the respective block diagram.

Algorithm for combining fragmented nuclei.

- Inputs: Stage-2 refined centroid image, 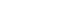.
- Output: Stage-3 or final centroid image, 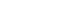.
- Assume a suitable distance threshold value, 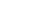.

1. Compute centroid coordinates from 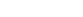.
2. For each centroid (*i*), compute all possible Euclidean distances, 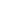 with other centroids (*j*) for 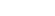 and 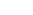

3. Create a list that contains *i* with other centroid indices (*j*), satisfying the condition: 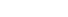

4. If there is no group that contains listed indices, create a new group with them. Otherwise, identify common groups having one or more listed elements and merge them into a new group, which must contain unique indices from common group and current list.
5. Continue step- 2 to step- 4 for all remaining centroids. This will result in unique index groups.
6. For each group, compute the final centroid by averaging the coordinates (*x*, *y*, *z*) of group members.
7. Create a 3D centroid image, 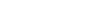 that contains the final centroids.

The above procedure combines fragmented nuclei and produces final nuclei centroids. We empirically fixed the value of the distance threshold to 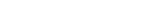 where 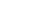 is the diameter of the largest nucleus, usually found in the lowest time–point image. In order to measure 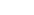 the preprocessed version of the lowest time point image is displayed in a visualization software. Alternatively, we can open the original image in the software and apply some kind of enhancement technique, for example smoothing and noise filtering. In our case, we applied 3D Gaussian smoothing and median filtering for the preprocessing and enhancement. We then applied a manual threshold that produced a binary image, in which the labeled regions indicate nuclei whereas the black (i.e., zero labeled) regions represent background. This binary image is then observed slice by slice. The slice, i.e., the x-y plane containing the largest single 2D object gives its center along the z-axis. We can then measure the nucleus (object) size in the x-y plane by moving the mouse and reading its positions around the object’s boundary. We can also automate the above procedure using a typical threshold technique, for example Otsu method [14]. After the binarization of the image, we can compute the largest single nucleus by checking sphericity of each connected component and counting its associated voxels.

## Results

In this section, we describe an experiment using 100 3D images and evaluate the performance of the proposed method. Both qualitative and quantitative results on the original 3D images are provided to demonstrate the potentiality of our method.

### Qualitative Evaluation of the Detection Results

**Figure 7:**
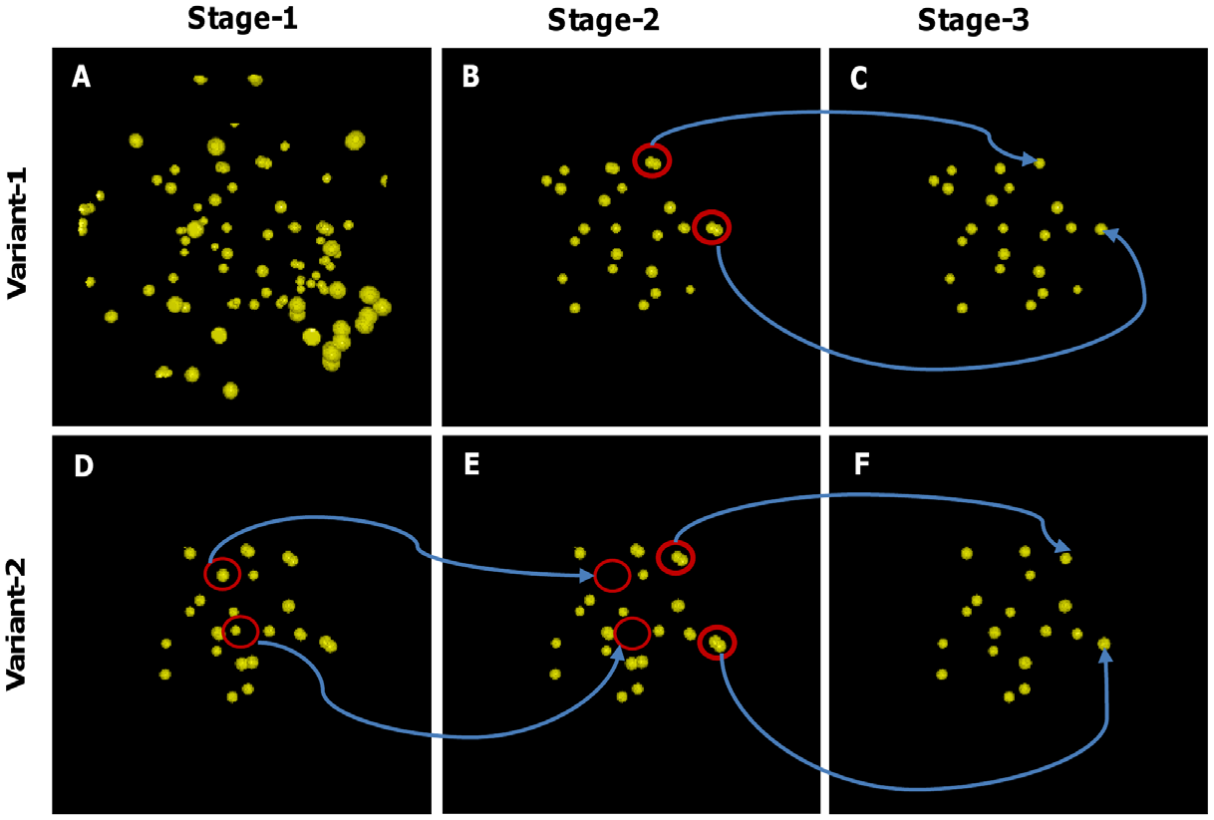
Results for centroid extraction at various stages of our method. (A, D) Stage-1 results of centroid extraction after local maxima searching for a sample image at t70, (B, E) Refined results of Stage-1 centroids after profile shape analysis using locally enhanced image (Stage-2), and (C, F) Refined results of Stage-2 centroids after combining fragmented nuclei (Stage-3). Top and bottom rows show the results for Variant-1 and Variant-2, respectively.

Proposed method performs nuclei centroids in three stages. Qualitative performances in each stage can be justified by observing Fig. 7 and Table 1. The top and bottom rows in this figure show the results of three stages for Variant-1 and Variant-2, respectively. Many spurious centroids (i.e., maxima clusters), especially in the background regions, were detected (Fig. 7 A) in case of the whole image processing in variant-1. We can also see how profile analysis of LEI at Stage-2 removes these unexpected centroids (Fig. 7 (B, E)). In contrast, Stage-1 results that we obtain by processing candidate regions in Variant-2 show fewer centroids (Fig. 7 D) as compared to the whole image counterpart. However, Stage-3 processing effectively combines fragmented nuclei (see thick red circles in Fig. 7 (B - C) and Fig. 7 (E - F)) to extract final centroids.

**Table 1:** Results for nuclei extraction at various stages of centroid extraction.

	Number of nuclei			
	Proposed Method with Variant-1 (*Variant-2*)			Ground truth
Image	Stage-1	Stage-2	Stage-3	Nuclei
t12	44 (*28*)	22 (*23*)	17 (*17*)	17
t14	25 (*21*)	20 (*20*)	17 (*17*)	17
t40	114 (*17*)	16 (*15*)	16 (*15*)	17
t70	98 (*26*)	26 (*24*)	22 (*18*)	20
t85	77 (*46*)	37 (*40*)	30 (*29*)	30
t97	80 (*31*)	29 (*27*)	27 (*25*)	33

Number of estimated nuclei at various stages of the proposed method. Proposed method uses threshold parameters (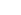 = 0.97) in the rough extraction stage (Stage-1), (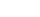 = 0.85) for the profile shape analysis (Stage-2), and (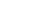  = 15 pixels) for merging fragmented nuclei (Stage-3).

**Figure 8:**
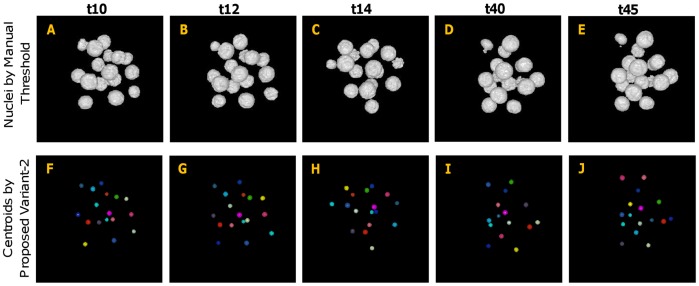
Results for centroid extraction by proposed method (Variant-2) for lower time–point images. (A–E) Preprocessed and manually thresholed 3D images (volume rendered) for time points t10, t12, t14, t40, and t45, respectively. (F–J) Corresponding results of centroid extraction. All individual centroids are represented by spherical regions using different colors.

**Figure 9:**
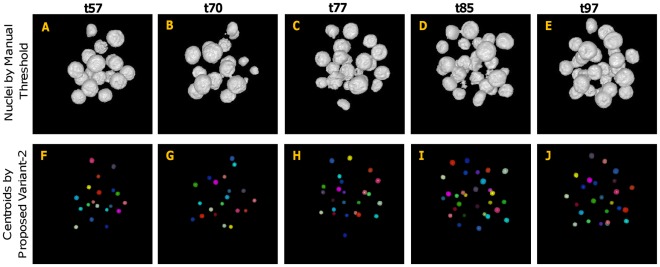
Results for centroid extraction by proposed method (Variant-2) for higher time–point images. (A–E) Preprocessed and manually thresholed 3D images (volume rendered) for time points t57, t70, t77, t85, and t97, respectively. (F–J) Corresponding results of centroid extraction. All individual centroids are represented by spherical regions using different colors.

In order to evaluate the centroid extraction results qualitatively, we applied the proposed method to mouse embryo images as described above. Several examples of centroid-extraction results, generated by the proposed method (Variant-2), are shown in Figs. 8 and 9. All estimated centroids are represented by small spheres, filled with different colors for easy visual inspection. The top row of this figure shows approximate object regions, which were obtained by setting manual threshold using preprocessed images followed by connected component labeling. The bottom rows in both figures show the results of the centroid–extraction by the proposed method. Despite the varying contrast and the inhomogeneity of the intensity structures, our proposed method has successfully extracted almost all the nuclei centroids, even if many of them are touching or close to each other. Similar analysis as above can also be done for Variant-1 of our method, but omitted here to avoid redundancy in contents. However, video Files S3, S4, S5, S6, S7, S8, S9, and S10 demonstrate the extraction results of both Variant-1 (Files S3, S4, S5, and S6) and Variant-2 (Files S7, S8, S9, and S10) for four 3D images corresponding to four different time points. The blue color in the clips indicates nuclei regions, obtained by an automatic threshold technique (Please refer to Pre-processing section for details), while red color indicates the extracted centroids by the proposed method.

**Figure 10:**
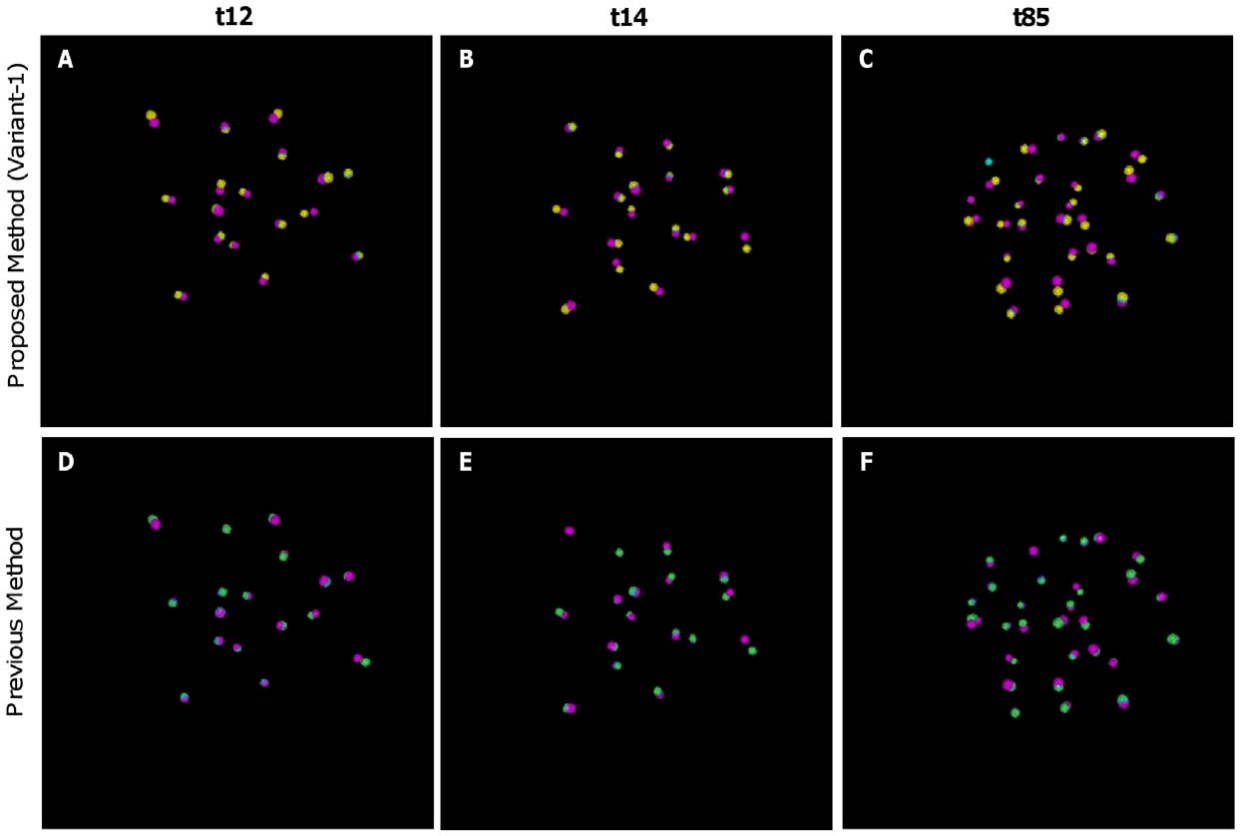
Visual comparison of estimated centroids with corresponding ground–truth centroids. Volume rendered view of the estimated and ground-truth centroids for images at time point (A, D) t12, (B, E) tp14, and (C, F) t85, respectively. Top row shows the results by our method, while bottom row shows the same by the method, proposed by Bao et al. Yellow and Green spheres show the estimated centroids by our and Bao’s methods, while pink spheres show ground–truth (GT) centroids. Blue color in the figures shows the overlapped regions between the estimated and GT centroids.

For visual comparison, an example of estimated results with corresponding GT centroids is also provided (see Figure 10); the yellow color in the top row and the green color in the bottom row indicate estimated centroids by the proposed (Variant-1) and an existing methods by Bao *et al.* [10], respectively. The pink color represents GT centroids, while blue color indicates overlapping of estimated and GT centroids. The closeness of the estimated and GT centroids in this figure qualitatively shows the competitive performance of our automatic method compared with the method by Bao *et al.* [10].

### Quantitative Evaluation and Performance Comparison

The evaluation of the experimental results is done by using ground truth data. Various evaluation metrics are used to quantify the detection accuracy, precision, and the error for the estimated positions of nuclei centroids.

#### Evaluation metrics

The quantitative performance of the proposed methods are analyzed by using the following metrics [17], [18]:
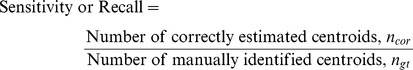
(12)

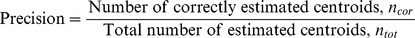
(13)

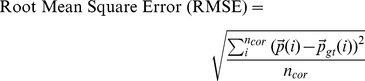
(14)In the above equations, 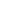 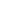 and 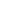 represent the correctly estimated nuclei or true positives, manually identified centroids or GT centroids, and the estimated total nuclei per 3D image, respectively. The position vectors 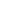 and 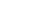 in Eq. 14 represent the estimated and GT coordinates of the nuclei centroids, respectively.

#### Computation of evaluation metrics

The above metrics are computed using the estimated and manually identified nuclei from each 3D image and the corresponding GT image, respectively. A local 3D spherical window with a radius of 10 voxels around each GT centroid is chosen to justify the availability of the estimated centroids. If any estimated centroid falls within the window volume, we consider it as a correct detection. If the window encloses more than one centroid, the one with the lowest distance is considered to be the correct detection. We can thus obtain a score of the total correct detection (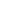) or true positives (*TP*) from each image. The false positives (*FP*), false negatives (*FN*), and the true negatives (*TN*) can also be obtained by finding estimated centroids inside or outside the window volume. However, we only need to compute 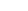 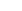 and 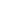 for the considered metrics since we already know true nuclei positions (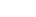) and numbers (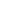) from GT data. To analyze the variability of metrics in a fixed duration, we used standard error, defined as 
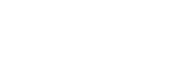
 where 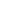 is a value of any of the above metrics at i-*th* time–point and 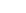 is the mean value of the series.

#### Analysis and comparison

Method proposed by Bao *et al.*: We compare the performance of our method with the method proposed by Bao *et al.* [10]. Their method was basically applied to *C. elegans* embryos, which roughly have regular cell dynamics [11]. At any given time, this method assumes approximately the same sized spherical nuclei, especially at early stages. At first, imaging data is preprocessed by low pass filtering followed by histogram-based threshold. This image is then convolved with a single scale cube filter having a kernel size same as the expected nuclear diameter at a given time point. This process produces a local signal, which was used to extract local maxima by choosing only one nucleus within a spatial range, fixed by the expected diameter. This step generates initial centroids, which were iteratively optimized in size and position to obtain final centroids. Some parameter values that we modified in conducting experiment with the Bao’s method are: (1) The start and end time points in the image series to be processed, i.e., 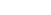 and 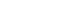 (2) The start and end planes in the stack to be processed, i.e., 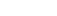 and 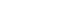 (3) Image voxel resolution, 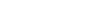 and 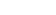 (4) Expected nuclei size 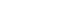 pixels, and the neighborhood size for the lowpass filter 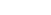 pixels. There are some other parameters related to noise threshold, the computation of spherical model, and scanning box algorithm for computing local maxima. Detail explanation of parameters will be found in [10].

**Figure 11:**
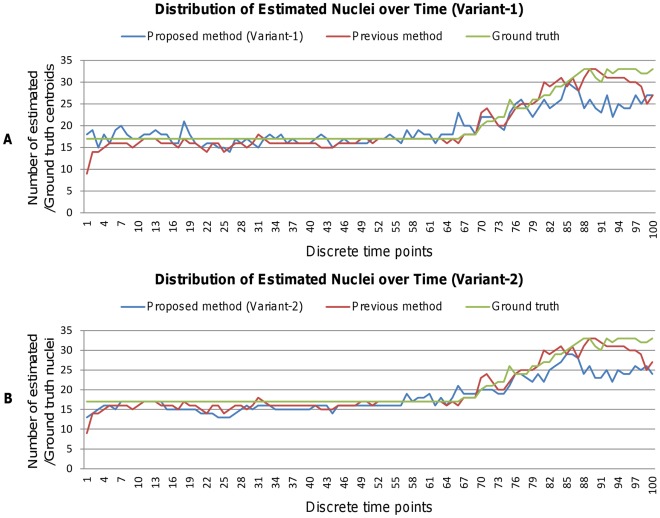
Results of number of estimated nuclei by our method. Blue and red graphs show the plots of the estimated nuclei for (A) Variant-1 and (B) Variant-2 of our method and Bao’s method, respectively. The green graph shows manually identified GT centroids. These plots involve 100 3D images, captured at 100 discrete time points in the early developmental period of mouse–embryo.

In order to compare the performance of the proposed method with Bao’s method, we conducted experiments using the same set of mouse imaging data. Figures 11 A and B show the results of nuclei detection. Blue and red curves in each figure indicate the number of estimated nuclei by the proposed (Variant-1 or Variant-2) and Bao’s methods, while the green curve shows GT centroids. Over majority time points, the proposed method, especially Variant-1 obtains closer estimates of centroid populations to GT centroids, compared to Bao’s method. These results suggest the biased behavior of Bao’s method especially for temporal analysis; because our method can stably supply more nuclei for establishing correspondences during cell tracking. The performance of Variant-2 in terms of the number of estimated nuclei looks similar to Bao’s method. Note that these graphs roughly reveal the performances without showing correctly estimated nuclei. More accurate analysis of the detection results can be done using metrics defined above.

**Figure 12:**
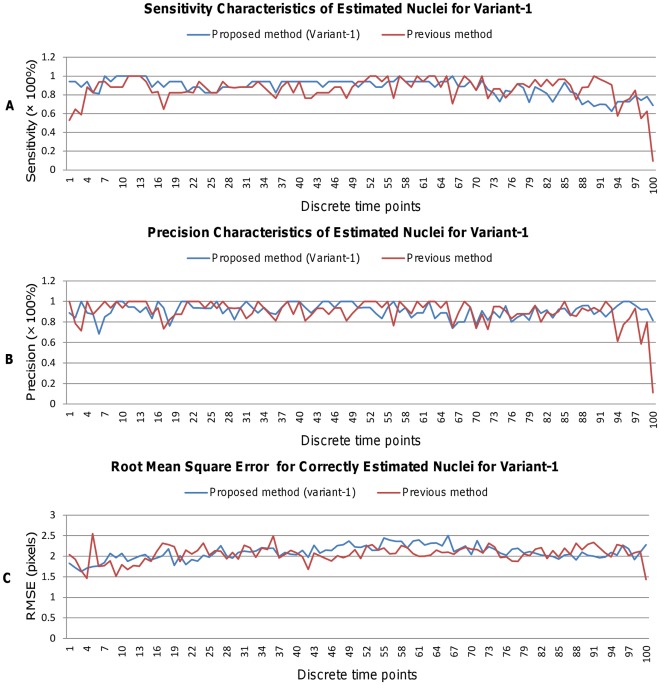
Comparison of Sensitivity, Precision, and RMSE metrics for estimated nuclei (Variant-1). Performance of nuclei detection over 100 time points (i.e., 100 3D images) in terms of (A) Sensitivity, (B) Precision, and (C) Root Mean Square Error (RMSE). Blue and red graphs show the performance curves for our proposed and Bao’s method, respectively.

**Figure 13:**
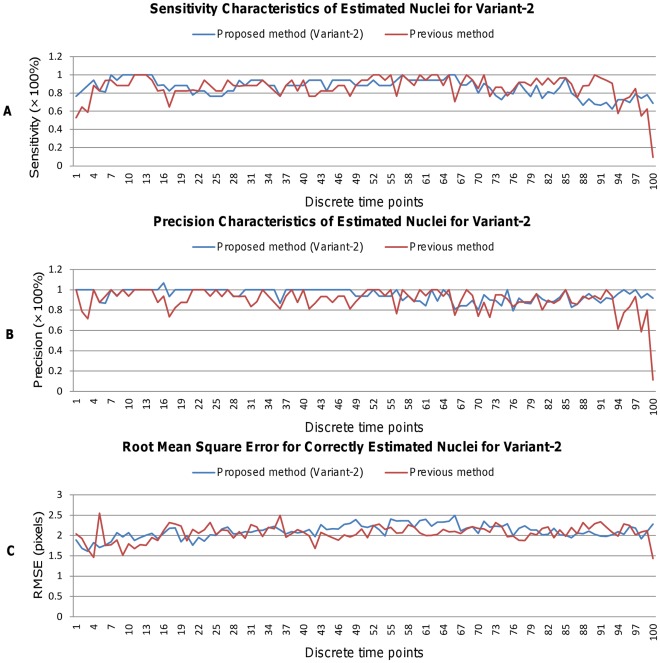
Comparison of Sensitivity, Precision, and RMSE metrics for estimated nuclei (Variant-2). Performance of nuclei detection over 100 time points (i.e., 100 3D images) in terms of (A) Sensitivity, (B) Precision, and (C) Root Mean Square Error (RMSE). Blue and red graphs show the performance curves for our proposed and Bao’s method, respectively.

Figures 12 and 13 show instantaneous values for (A) sensitivity, (B) precision, and (C) root–mean–square error (RMSE) for variant-1 and variant-2, respectively. In general, higher sensitivity and precision with a slightly larger RMSE are obtained by our method as compared to Bao’s method. Although sensitivity decreases a bit after time point t66, precision maintains high values even after t66. Above figures also indicate that Variant-1 obtains higher instantaneous sensitivity than Variant-2 and vice versa for the precision. This is because full image processing in Variant-1 allows the extraction of low–contrast nuclei that Variant-2 may miss during the processing of candidate regions at the cost of little increased false positives. However, relatively fewer fluctuations in performance dynamics (i.e., sensitivity, precision, and RMSE) indicate the effectiveness of our method.

**Figure 14:**
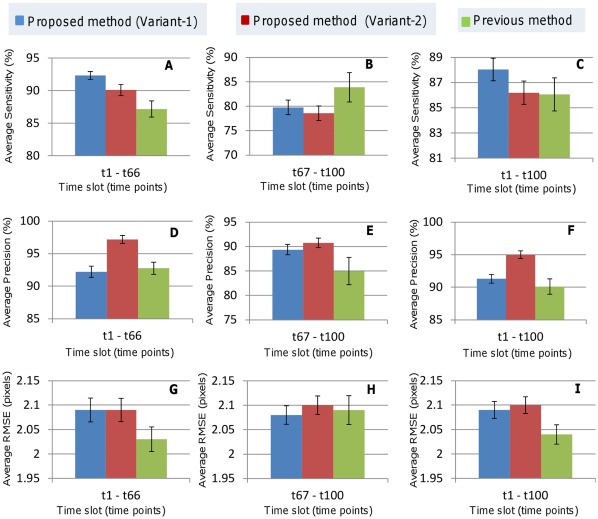
Results for average sensitivity, precision, and RMSE. (A – C) Average sensitivity, (D – F) Average precision, and (G – I) Average RMSE for image series (t1– t66), (B) (t67– t100), and (C) (t1– t100), respectively. Series (t1– t66) and (t67– t100) indicate fixed and variable number of nuclei, while series (t1– t100) indicates the whole dataset. Blue, red, and green bars show the average performances with standard error bars for the proposed method with (i) Variant-1, (ii) Variant-2, and the previous method, respectively.

More quantitative analysis can be performed from Table 2 and Figure 14. They include average metric values corresponding to silent (t1–t66) and active (t67–t100) states including whole time series (t1–t100). In the silent state, the proposed method shows much better mean sensitivity 92.32% (Variant-1) or 90.11% (Variant-2) compared to Bao’s method (87.17%) (Fig. 14 A). But in the active state, it shows smaller mean sensitivities (i.e., 79.74% for Variant-1; 78.59% for Variant-2) than that (83.91%) by Bao’s method (Fig. 14 B). One reason for this lower performance in the active stage is the lack of flexibility in multiscale filtering. We used fixed set of parameters for the entire series of images. But the parameters that produce better enhancement, i.e., higher sensitivities for lower time–point images may cause inappropriate enhancement, i.e., lower sensitivities to higher time–point objects (nuclei) because of their smaller sizes. Future work has to be done to resolve this problem. Figure 14 H shows that variant-1 and variant-2 of our method produce similar position errors in the active state (2.08 and 2.10 pixels) to Bao’s method (2.09 pixels). But they obtain slightly larger errors in the silent state (2.09 and 2.09 pixels) as well as for whole series (2.09 and 2.10 pixels) than those (2.03 and 2.04 pixels) obtained by Bao’s method (Fig. 14 (G, I)). It seems that above increase in the average RMSE may be eliminated by adjusting parameters for multiscale filtering. However, the proposed method attains comparable or better precision in the silent as well as active states (i.e., 92.21% and 89.35% for Variant-1; 97.18% and 90.75% for Variant-2) compared to Bao’s method (92.77% and 84.97%), respectively (Fig. 14 (D, E)).

**Table 2:** Overall detection performances of proposed method.

Metrics	Average performance comparison					
(Averagevalue of metrics)	Proposed method with Variant-1 (*Variant-2*)			Previous method (Bao et al.)		
	t1– t66	t67– t100	t1– t100	t1– t66	t67– t100	t1– t100
Sensitivity(%)	92.32(*90.11*)	79.74(*78.59*)	**88.04(** ***86.19*** **)**	87.17	83.91	86.06
Precision (%)	92.21(*97.18*)	89.35(*90.75*)	**91.30 (** ***95.00*** **)**	92.77	84.97	90.11
RMSE (pixels)	2.09(*2.09*)	2.08(*2.10*)	**2.09 (** ***2.10*** **)**	2.03	2.09	2.04

Total time span (t1 to t100) is divided into two slots corresponding to fixed (t1 to t66) and variable (t67 to t100) number of cells, and average metric scores were obtained by averaging scores in each time slot.

Averaging over entire dataset shows that the proposed method achieves an average sensitivity of 88.04% (Variant-1) or 86.19% (Variant-2), which is 1.98% or 0.13% higher than that with Bao’s method (86.06%) (Fig. 14 C). Our method also obtains 91.30% (Variant-1) or 95.00% (Variant-2) average precision, which is 1.19% or 4.89% higher than that with Bao’s method (90.11%) (Fig. 14 F). Whatsoever, the proposed method shows relatively stable performance, i.e., smaller standard error as compared to Bao’s method (see error bars in Fig. 14).

### Computational Efficacy, Hardware, and Software

We implemented the proposed method using Microsoft Visual Studio 2008 for the windows platform. Most of the source codes were written using visual C++ except few cases, where we used some functions from the library, entitled Media Integration Standard Toolkit (MIST), which is freely available in the [19]. The visualization of 3D images and the making of video clips were done using freely available software “ImageJ” [20] and another non-free software “PLUTO” [13]. Source code is not open at the moment. Tests were executed using a windows PC having an Intel(R) Core(TM) i7 CPU 3.20GHz with 8GB RAM. Our cell detection program takes about 0.5–3 minutes to process a 3D image of size 261×261×224 with 2D slices as output. Although, the processing of candidate regions has computational advantages (about 0.5 minutes per image), it may miss low contrast nuclei in the worst case. On the other hand, the whole image processing in Variant-1 ensures the extraction of low contrast centroids at the cost of computational time (about 3 min per image).

## Discussion

The major bottleneck in the detection of nuclei centroids from 3D mouse embryo images is the juxtaposed nature of cell populations even in the early stage of the development. In our method, we addressed this problem by directly computing nuclei centroids without knowing their actual sizes and shapes. Typical methods require advanced segmentation techniques to extract cell nuclei and their centroids accurately. The accuracy usually falls when segmentation accuracy is poor as a result of noisy data or algorithmic limitations. They often fail when cells are closely located and/or when many of them are dividing. In contrast, our method extracts nuclei regions as local maxima that characterizes them well. An average sensitivity of 88.04% by our method (Variant-1) indicates the efficiency of nuclear detection.

The use of candidate regions (Variant-2) confines processing to a small number of object-enclosing voxels of the hybrid image and hence reduces the computation cost. Moreover, by processing candidate regions, we can avoid many false local maxima that usually appear in the background regions due to noise components. The processing load and the detection error (if any) of the Stage-2 is also reduced as it has to process fewer objects before progressing to Stage-3. We used Otsu method [14] for the extraction of the candidate regions. One reason of choosing Otsu method is its simplicity to apply with the higher dimensional (2D/3D) images. Within the given degree of non-uniform illuminations, it works well with the confocal fluorescence images and we obtain 86.19% sensitivity and 95% precision for the used dataset. However, Otsu method may miss low contrast nuclei in the worst case when the effects of noise or non-uniform illuminations become severe. One way that may improve our results is to perform homomorphic filtering [21] before applying Otsu method. This filter non-linearly maps the intensity images into logarithmic domain, where simple linear filtering techniques can be used to reduce the effects of non-uniform illuminations. Another alternative is to try a method that is robust against non-uniform illuminations [22].

There are mainly six parameters in our method. The first three i.e., 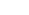 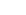 and 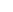 were used for computing the lengths for multiscale filters. To get benefits from such filtering, these lengths should approximately cover the sizes of all nuclei in a 3D image. We empirically selected them based on the largest nucleus diameter as explained in the “method” section. The rest three parameters are related to the Stage-1, Stage-2, and Stage-3 of the centroid extraction technique. The ratio threshold (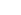) controls the production of initial candidate centroids. The profile-shape threshold (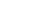) removes false centroids from the initial detection results. The distance threshold (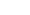) combines fragmented nuclei. Since an ideal nucleus is supposed to have gradually falling intensity profile from its center, the ideal values of the 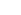 or 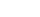 will be 100%. A typical selection of 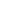 value usually creates point-clusters as representatives of candidate nuclei. If we increase its value, the size of the cluster reduces. Since the selection of a single or a few points is sufficient to represent a nucleus, we can select a high 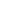 value in principle. However, depending on the SNR level in an image, we can reasonably choose 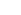 that is closer to the ideal value so that no object remains undetected in Stage-1. The selection of a very high value for the 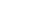 is not recommended as we may miss some nuclei because of their complex or asymmetric shapes, i.e., low shape score (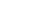) as compared to the threshold value. Therefore, a value that is usually smaller than that of the 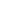 could be a reasonable choice for 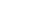. In our study, we chose 97% for the 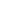 and 85% for the 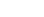 On the other hand, the selection of 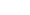 should be such that the search-length for finding nuclei fragments approximately covers the volume of each object in an image. Nevertheless, we can select above parameters in a flexible manner, because sequential processing in a stage takes care of the results in the previous stage.

The selection of Stage-3 threshold, i.e., 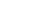 is made using 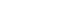
%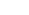, where 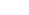 value is determined in an interactive manner as detailed in the “Method” section. In principle, it is better to have slightly decreasing value of the distance threshold to process objects (nuclei) after each division, because we observe a slight decrease in the average nucleus size over the process of cell division. However, the selection of threshold value is not much sensitive to the number of cell divisions because a few intra-nucleus micro-structures, i.e., fragments are occasionally visible and they appear in the interior part of the nucleus. With the verified case of up to 33 nuclei, we found that a fix value of threshold is sufficient to obtain reasonably high precision and sensitivity that we have already achieved. We assumed that the nuclei are spherical objects. Since there are multiple objects of different sizes and shapes in an image, an ideal value for this threshold (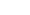) should be adaptive and equal to the radius of each nucleus. Without perfect segmentation, it is impossible to know object sizes and shapes, especially for juxtaposed or overlapped objects. Therefore, we chose above threshold as a rule of thumb so that we can mostly cover all objects (i.e., nuclei) during fragment searching for their merging. This simplification relaxed us adopting any segmentation and optimization techniques for object extraction. The presence of the extra nuclear materials produces a sufficient gap between two nuclei, which helped us fixing this kind of empirical threshold without committing much error.

We perform the experiment using images, indexed from t1 to t100. Cell populations remain fixed to 17-cells until time–point t66, after which they increase quickly and reaches to 33-cells at t100. It is observed that the detection performance decreases slightly (see Figures 12 and 13 - (A)), when cell population increases after time–point t66. Average RMSE is also increased slightly (2.09 pixels for Variant-1, 2.10 pixels for Variant-2 and 2.04 pixels for Previous method). Current setting of multiscale parameters is done empirically by knowing the diameter of the largest nucleus corresponding to the lowest time–point image. However, accuracy may be improved by computing multiscale parameters adaptively over time. Future research will be conducted to improve detection performance at higher time points.

We have designed our method for processing higher (three or more) dimensional images. In general, our method has no limitations to apply for more complex structures, cell confluency or high degree of cell overlapping in an X-Y plane. In our current dataset, we have 17 to 33 cells and it has a certain degree of complex structures or cell overlapping. With this dataset, we have obtained an average sensitivity of 88.04% by Variant-1 and 86.19% by Variant-2 and average precision of 91.30% by Variant-1 and 95.00% by Variant-2. However, as almost all image-processing algorithms does, the applicability of our method depends on the signal to noise ratio (SNR) and the effective spatial resolution of voxels in imaging data. It is quite natural with the current technique for fluorescence imaging because signal to noise ratio becomes low when cell grows in numbers. At high time points, a large fraction of the emitted light is scattered and contributes to the background intensities. Therefore, the spatial resolution which is suitable at the lower time points is no longer sufficient to image larger or complex cell populations at higher time points. As long as the imaging techniques preserve the true signatures of cells in the intensity domain, our methods could be applied even if there is heterogeneity in the cell shapes. Our method usually performs efficient cell detection using a fixed set of parameters. Of course, adaptive parameter setting especially for multiscale filtering may provide some additional advantages in terms of detection accuracy.

Similarly, our method may also be applied to investigate embryogenesis later than morula stage if both SNR and spatial resolution of data are sufficient. In the blastula stage, cells form a hollow sphere as they mainly reside in its outer surfaces. Two situations might occur: (1) Low scattering effects due to the absence of deep layer cells. (2) Low signal to noise ratio due to higher cell density. Therefore, the imaging technique should be able to trade off these opposing situations. Within this limitation, if the imaged signatures are sufficient to preserve the cell identity, our method can be applied to analyze blastula stage of embryogenesis. Of course, the degree of the detection accuracy depends on the image quality, cell density, and the parameter setting of the method. We will perform this analysis as our future work.

We have already seen that the sensitivity decreases a bit with the increase of cell populations even though the precision remains high. Although not included, we analyzed the specificity i.e., 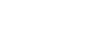 where *TN* and *FP* represent the true negative and false positive, of our method using 4D imaging data. Since we have 17 to 33 cells per image and they are represented by their centroid voxels, the ground truth negative is quite high for a given 3D image of size 261×261×224. As a result, the estimated *TN* is also significantly larger than the estimated *FP* irrespective of the methods. This leads to a very high specificity, usually 99% or higher for the proposed and the previous method.

### Conclusion

A novel automated method is proposed for the identification of nuclei centroids from fluorescence microscopy images. Two variations of the method, where Variant-1 uses whole 3D images and Variant-2 uses candidate regions for later processing, are discussed. A 3D Gaussian filter followed by a 3D median filter is first applied for smoothing and noise reduction. A multiscale cube filtering is then adopted for local enhancement of whole image or candidate regions. A three stage procedure for centroid extraction is then suggested. Stage-1 processing generates candidate centroids by using threshold on characteristic ratio *R* at every voxel. Stage-2 processing removes spurious centroids from Stage-1 results by analyzing shape score for intensity profiles. Since Stage-2 results may contain fragmented nuclei, an iterative procedure is proposed to combine them. An experiment with 100 3D images shows an improvement of performance of our method in terms of average sensitivity (0.13% to 2.0%) and precision (1.19% to 4.89%) as compared to Bao’s method, originally proposed for analyzing *C. elegans* embryos. However, the proposed method obtains a slightly larger average RMSE (0.05 pixels to 0.06 pixels) as compared to the previous method. Accuracy may be improved by computing multiscale parameters adaptively over time. Future works will be directed to solve these issues and to extend the proposed method for tracking cell populations.

### Supporting Information

**File S1 – Documentation file**. This file is uploaded as a pdf file (i.e., “usage.pdf”). It provides descriptions of the supplementary image and video files (i.e., files S2 to S10) and explains how to view their contents.

**Files S2 to S10 – Image and video files**. File S2 is a video clip, which contains a set of contrast-enhanced 2D images of mouse embryonic cells. Video clips in Files S3, S4, S5, and S6 demonstrate the centroid extraction results from Variant-1, while the same in Files S7, S8, S9, and S10 represent the corresponding results from Variant-2 of the proposed method. Four 3D images corresponding to different time points are used to obtain these results.

## Supporting Information

File S1 **This file provides an explanation about other supplementary files.** It describes the contents of the video clip Files S2 to S10 and the procedure of displaying their contents.
(PDF)

File S2 **This file shows the enhanced version of an original 3D image.** An original 3D image that corresponds to time point t10 is histogram equalized for the easy visualization of the nuclei objects in the image.
(AVI)

File S3 **This file shows the centroid extraction results that was produced by the Variant-1 of the proposed method.** It represents 3D images corresponding to time point t20. The red spheres indicate the estimated centroids, while the blue color indicates approximate nuclei regions around each centroid. Some non-object background slices from each end of the z-axis were removed before making the clips.
(AVI)

File S4 **This file shows the centroid extraction results that was produced by the Variant-1 of the proposed method.** It represents 3D images corresponding to time point t40. The red spheres indicate the estimated centroids, while the blue color indicates approximate nuclei regions around each centroid. Some non-object background slices from each end of the z-axis were removed before making the clips.
(AVI)

File S5 **This file shows the centroid extraction results that was produced by the Variant-1 of the proposed method.** It represents 3D images corresponding to time point t45. The red spheres indicate the estimated centroids, while the blue color indicates approximate nuclei regions around each centroid. Some non-object background slices from each end of the z-axis were removed before making the clips.
(AVI)

File S6 **This file shows the centroid extraction results that was produced by the Variant-1 of the proposed method.** It represents 3D images corresponding to time point t92. The red spheres indicate the estimated centroids, while the blue color indicates approximate nuclei regions around each centroid. Some non-object background slices from each end of the z-axis were removed before making the clips.
(AVI)

File S7 **This file shows the centroid extraction results that was produced by the Variant-2 of the proposed method.** It represents 3D images corresponding to time point t20. The red spheres indicate the estimated centroids, while the blue color indicates approximate nuclei regions around each centroid. Some non-object background slices from each end of the z-axis were removed before making the clips.
(AVI)

File S8 **This file shows the centroid extraction results that was produced by the Variant-2 of the proposed method.** It represents 3D images corresponding to time point t40. The red spheres indicate the estimated centroids, while the blue color indicates approximate nuclei regions around each centroid. Some non-object background slices from each end of the z-axis were removed before making the clips.
(AVI)

File S9 **This file shows the centroid extraction results that was produced by the Variant-2 of the proposed method.** It represents 3D images corresponding to time point t45. The red spheres indicate the estimated centroids, while the blue color indicates approximate nuclei regions around each centroid. Some non-object background slices from each end of the z-axis were removed before making the clips.
(AVI)

File S10 **This file shows the centroid extraction results that was produced by the Variant-2 of the proposed method.** It represents 3D images corresponding to time point t92. The red spheres indicate the estimated centroids, while the blue color indicates approximate nuclei regions around each centroid. Some non-object background slices from each end of the z-axis were removed before making the clips.
(AVI)
