# Supplementary material for: Automatic Extraction of Nuclei Centroids of Mouse Embryonic Cells from Fluorescence Microscopy Images
Source: PLoS One. 2012 May 8;7(5):e35550. doi: 10.1371/journal.pone.0035550 (PMC3348125; doi:10.1371/journal.pone.0035550)
Supplement: File S1 — This file provides an explanation about other supplementary files. It describes the contents of the video clip Files S2 to S10 and the procedure of displaying their contents. (PDF) [file pone.0035550.s001.pdf]

Instructions of Using Supplementary Files

Following are the image and video files included as supplementary information:

- Sample video clip of the imaging data

1. S2 File (t10.avi)—Video clip for enhanced 2D slices

- Centroid Extraction Results from the proposed Variant-1

2. Supplementary files S3, S4, S5, and S6 are uploaded as t20v1.avi, t40v1.avi, t45v1.avi, and t92v1.avi, respectively.

- Centroid Extraction Results from the Proposed Variant-2

3. Supplementary files S7, S8, S9, and S10 are uploaded as t20v2.avi, t40v2.avi, t45v2.avi, and t92v2.avi, respectively.

In our experiment, we used 100 3D volume images that represent the early stages of mouse embryogenesis. These images were captured in the form of .tif format using fluorescence microscopy technique. Above files show a set of 2D sample images, and some sample results by our method. All .avi files except "t10.avi" represent results for nuclei centroid extraction. It shows a set of contrast enhanced 2D sample images. Since original images are quite black and their contents are not visible, we performed histogram equalization so that image contents are approximately viewable.

"t (number)" in the above files indicates time point and v1 or v2 represents the Variation-1 or Variation-2 of our proposed method described in this paper. The output 2D slices by our method were generated in the ".jpg" format. These slices were saved as "avi" files using ImageJ software. Original 3D images have 28 z-slices per image, while the outputs have 224 slices per image, because we use 8 times interpolation. However, some top and bottom slices do not contain any objects (nuclei). We therefore remove them before generating ".avi" movie clips. The blue regions in the image are approximate nuclei regions, obtained by an automatic threshold technique (Please see our paper for detail), while red colors indicate the extracted centroids that we obtain by our proposed method.

Above video clips can be viewed by using Windows media player. If you face any problem while viewing, please contact us in the following address:

Md. Khayrul Bashar,
Assistant Professor,
Institute of Industrial Science,
University of Tokyo, Japan
Email: khayrul_b@yahoo.com
Phone and Fax: +81-03-5452-6797
